# Development of Air Cell System Following Canal Wall Up Mastoidectomy for Pediatric Cholesteatoma

**DOI:** 10.3390/jcm13102934

**Published:** 2024-05-16

**Authors:** Yusuke Yamada, Akira Ganaha, Nao Nojiri, Takashi Goto, Kuniyuki Takahashi, Tetsuya Tono

**Affiliations:** 1 Department of Otolaryngology, International University of Health and Welfare, Tochigi 324-8501, Japan; y-yamada@iuhw.ac.jp (Y.Y.); nao-nojiri@iuhw.ac.jp (N.N.); tono@med.miyazaki-u.ac.jp (T.T.); 2 Department of Otolaryngology and Head and Neck Surgery, Faculty of Medicine, University of Miyazaki, Miyazaki 889-1692, Japan; takashi_goto@med.miyazaki-u.ac.jp (T.G.); kuniyuki_takahashi@med.miyazaki-u.ac.jp (K.T.); 3 Department of Otorhinolaryngology, Head and Neck Surgery, International University of Health and Welfare, Narita Hospital, Chiba 286-0124, Japan

**Keywords:** air cell development, canal wall up mastoidectomy, pediatric middle ear cholesteatoma, postoperative pneumatization, temporal bone CT

## Abstract

**Background:** The development of temporal bone pneumatization is related to the postnatal middle ear environment, where the development of air cells is suppressed with otitis media in early childhood. However, whether air cell formation restarts when mastoidectomy is performed during temporal bone pneumatization remains unclear. Herein, we evaluated temporal bone pneumatization after canal wall up (CWU) tympanomastoidectomy for middle ear cholesteatoma in children. **Methods:** In total, 63 patients, including 29 patients with congenital cholesteatoma (CC) and 34 patients with acquired cholesteatoma (AC), were assessed using a set of pre- and postoperative computed tomography images. The air cells of the temporal bone were divided into five areas: periantral (anterior), periantral (posterior), periantral (medial), peritubal, and petrous apex. The number of areas with air cells before and after surgery was compared to evaluate temporal bone pneumatization after surgery. **Results:** A total of 63 patients, comprising 29 with CC and 34 with AC (pars flaccida; 23, pars tensa; 7, unclassified; 4), were evaluated. The median age of patients (18 males and 11 females) with CC was 5.0 (range, 2–15 years), while that of the AC group (23 males and 11 females) was 8 (range, 2–15 years). A significant difference in air cell presence was identified in the CC and AC groups after surgery (Mann–Whitney U, *p* < 0.001 and *p* = 0.003, respectively). Between the two groups, considerably better postoperative pneumatization was observed in the CC group. A correlation between age at surgery and gain of postoperative air cell area development was identified in the CC group (Spearman’s rank-order correlation coefficient, r = −0.584, *p* < 0.001). In comparison with the postoperative pneumatization rate of each classified area, the petrous apex area was the lowest in the CC and AC groups. **Conclusions:** Newly developed air cells were identified in the temporal bones after CWU mastoidectomy for pediatric cholesteatoma. These findings may justify CWU tympanomastoidectomy, at least for younger children and CC patients, who may subsequently develop air cell systems after surgery.

## 1. Introduction

Development of the temporal bone air cell system begins at 21–22 weeks of gestation originating at aditus ad antrum, with the mastoid antrum reaching completion around 33 weeks of gestation [1]. At birth, the mastoid cavity expands as embryonic cells in the middle ear cleft are rapidly absorbed and replaced [2], and temporal bone pneumatization typically concludes by the age of puberty [1,3]. The development of temporal bone pneumatization is influenced by the postnatal middle ear environment, with air cells potentially suppressed by otitis media in early childhood or following inadequate otologic surgery [4]. While tympanic ventilation tube insertion and otitis media treatment can restore pneumatization [5], there is a lack of studies investigating whether the air cell system reforms after mastoidectomy during temporal bone pneumatization.

The primary objective of canal wall up (CWU) tympanomastoidectomy is to establish an aerated middle ear cavity after eradication of middle ear pathologies. However, our experience from second-look operations and/or postoperative CT scans reveals considerable variability in mastoidectomy cavity conditions among patients. Contrary to our expectation at that time that the middle ear space should be aerated after tympanomastoidectomy, only one-third of cholesteatoma cases exhibited repneumatization of the mastoidectomy cavities [6]. Subsequent studies indicate repneumatization is more prevalent in younger age groups than older ones [7], with some patients even developing new air cells, suggesting ongoing pneumatization despite mastoidectomy in children. Although pneumatization following surgery for inflammatory middle ear diseases correlates with the primary disease or nonoperated contralateral ear [6], the reactivation of air cell formation following mastoidectomy remains unclear. This study aimed to provide evidence of newly formed air cell systems following CWU tympanomastoidectomy for pediatric cholesteatomas. To this end, we retrospectively evaluated CT images obtained before and after surgery in a limited number of pediatric patients who underwent consecutive CT examinations.

## 2. Materials and Methods

Postoperative patients aged 15 years or younger at the time of initial CWU tympanoplasty for middle ear cholesteatoma at the University of Miyazaki Hospital since 1988 were studied retrospectively. Patients with both preoperative and postoperative CT images acquired at least 5 years after the initial surgery were included. CT scans had been conducted one month before surgery and 12 months postsurgery. Follow-up CTs more than 5 years after the initial surgery had been performed for patients with a stable tympanic membrane before referral out of the university hospital. Consequently, all subjects in the study had no pathological otoscopic findings such as perforation, middle ear effusion, or retraction of the reconstructed tympanic membrane. The inclusion and exclusion criteria are presented in Table 1. Patients were classified and staged according to the criteria for middle ear cholesteatoma proposed by the Japan Otological Society [8], allowing the use of the PATM system for locating cholesteatoma extension into four sites (P: protympanum, T: tympanic cavity, A: attic, and M: mastoid) of the middle ear space. The extent of cholesteatoma was defined as the total number of cholesteatoma extension sites. Data were analyzed using SPSS statistical package (SPSS for Windows version 26; SPSS Inc., Chicago, IL, USA). *p*-values < 0.05 were considered statistically significant. This study was conducted with approval from the ethics committee of the University of Miyazaki (registration number: O-0751).

### 2.1. Surgical Technique

CWU tympanomastoidectomy in these patients began with mastoidectomy to eliminate atticoantral pathologies through a postauricular incision. Mastoid drilling was typically limited to the cholesteatoma’s extent to preserve mucosalized air cells as much as possible. The communication route between the epitympanum and the eustachian tube was routinely established via the supratubal recess, known as anterior tympanotomy [9]. Posterior tympanotomy was also performed when necessary to access cholesteatoma extending into the posterior part of the tympanic cavity. Defects in the scutum were always reinforced with one or two pieces of sliced auricular cartilage. None of the patients in this series underwent mastoidectomy cavity obliteration.

Second-look procedures were conducted in 25 patients 12 months after the initial surgery to remove residual cholesteatoma either through the mastoidectomy cavity or via a transcanal approach, along with ossiculoplasty if necessary.

### 2.2. Image Evaluation

**Figure 1:**
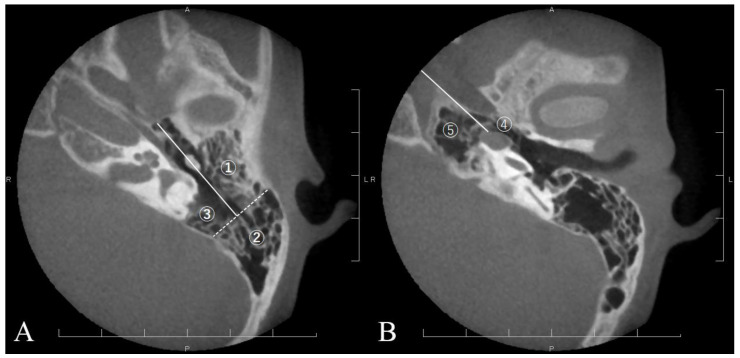
Axial CT images in the left temporal bone, indicating five regions of pneumatized air cell systems by the white line (**A**): level of the atticoantrum, showing the anterolateral area of periantral cells (①), posterolateral area of periantral cells (②), and medial area of periantral cells (③). The white solid line indicates the longitudinal axis of the atticoantral cavity. The white-dashed line is orthogonal to the longitudinal line at posterior end of mastoid antrum. (**B**) Level of the tympanic cavity, showing peritubal cells (④) and petrous apex cells (⑤). The white solid line connecting the inside of the carotid artery with otic capsule divides the two areas.

**Figure 2:**
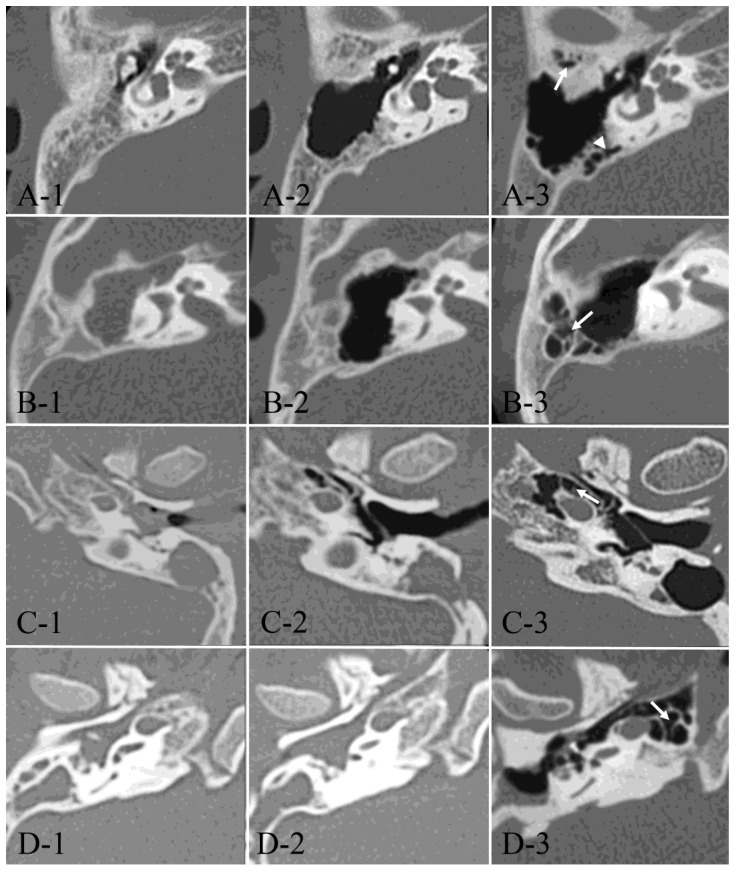
Developmental process of postoperative pneumatization. Axial CT images from four typical cases (**A**–**D**) at preoperative, one year postoperative, and the last follow-up examinations (**1**–**3**). (**A-1**–**A-3**): A 9-year-old male with right-sided AC showed postoperative development of the anterolateral area of periantral cells (arrow) and the medial parts of periantral cells (arrowhead). At the age of 14. (**B-1**–**B-3**): A 5-year-old female with right-sided AC showed postoperative development of the posterolateral area of periantral cells (arrow) at the age of 13. (**C-1**–**C-3**): A 6-year-old male with left-sided AC showed postoperative development of peritubal cells (arrow) at the age of 22. (**D-1**–**D-3**): A 5-year-old male with right-sided AC showed postoperative development of petrous apex cells (arrow) at the age of 24.

High-resolution CT studies were conducted on a GE 8800 or 9800 scanner (General Electric, Milwaukee, WI, USA), utilizing 1.5 mm contiguous sections in semiaxial projection (OM line −30°), or with a slice thickness of 0.5 mm reconstructed from axial CT images (SOMATOM Definition AS+, SIEMENS, Erlangen, Germany). The degree of air cell development was assessed via CT scans using axial projections taken within 1 month before the initial surgery and at least 5 years postsurgery. Temporal bone air cells were categorized into five areas as follows: (1) anterolateral area of periantral cells: anterior to the long axis of the antrum; (2) posterolateral area of periantral cells: posterior to the long axis of the antrum; (3) medial area of periantral cells: medial to the long axis of the antrum or sino-dural area; (4) peritubal: areas in contact with the eustachian tube and tympanic opening; and (5) petrous apex: medial to the labyrinthine capsule (Figure 1). An otologist evaluated all CT images without knowledge of the otologic examinations and surgical methods manually. The number of areas with air cells before and after surgery were compared to evaluate temporal bone pneumatization development postsurgery. The typical developmental process of postoperative pneumatization for each area is depicted in Figure 2.

## 3. Results

Out of the 82 patients followed-up by with CT for over five years post initial CWU surgery, 19 patients were excluded due to motion artifact or insufficient field of CT scanning. Consequently, a total of 63 patients, comprising 29 congenital cholesteatoma (CC) and 34 acquired cholesteatoma (AC) cases (pars flaccida; 23, pars tensa; 7, unclassified; 4), were evaluated using pre- and postoperative CT images. The median age of patients (18 males and 11 females) with CC was 5 (range, 2–15) years, while that of patients in the AC group (23 males and 11 females) was 8 (range, 2–15) years. The age at surgery of the AC group was higher than that of the CC group. A Mann–Whitney test indicated that this difference was statistically significant (U = 692.50, z = 2.763, *p* = 0.006). The duration of follow-up in the CC and AC group was 87 (47–173) months and 88 (48–346) months, respectively. A Mann–Whitney test indicated that this difference was not statistically significant (U = 519.50, z = 0.366, *p* = 0.715). Demographic data are presented in Table 2.

Fisher’s exact test was used to compare the proportion of stage and extent of cholesteatoma between the CC and AC groups. No significant differences were identified in these characteristics between the two groups (Table 2). There was no correlation between the extent of cholesteatoma and age at surgery in the CC group (Spearman’s rank-order correlation coefficient, r = 0.064, *p* = 0.742) or in the AC group (r = −0.287, *p* = 0.100). In addition, no correlation was found between stage and age at surgery in the CC (r = −0.066, *p* = 0.733) or AC groups (r = −0.311, *p* = 0.074).

The number of pneumatized areas defined in this study (Figure 1) was compared between preoperative and long-term (over 5 years) post-mastoidectomy in the CC and AC groups (Table 2). The number of pneumatized areas before and after surgery in the CC group and AC group were presented in Table 3. A Wilcoxon signed-rank sum test indicated that there was a statistically significant increase in pneumatization area after surgery in both groups (Table 3). The numbers of air cell areas were compared between the CC and AC groups to evaluate the difference in the degree of temporal bone pneumatization by the type of cholesteatoma. The number of preoperative pneumatization areas in CC group (Mdn = 3) was higher than that in the AC group (Mdn = 2). A Mann–Whitney test indicated that this difference was statistically significant (U = 333.50, z = −2.245, *p* = 0.025). The number of postoperative pneumatization areas in the CC group (Mdn = 4) was higher than that in the AC group (Mdn = 3). A Mann–Whitney test indicated that this difference was statistically significant (U = 221.50, z = −3.876, *p* < 0.001) (Table 3).

**Figure 3:**
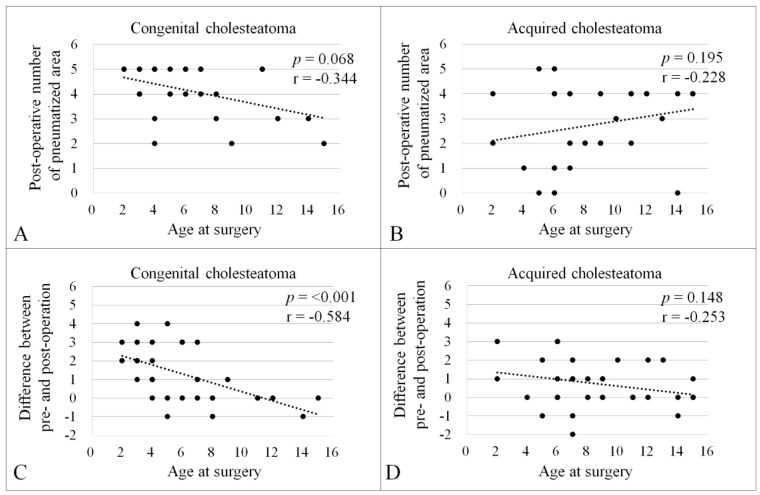
Correlation between the age at surgery and the number of pneumatized areas after surgery. Spearman’s rank-order correlation coefficient was conducted to evaluate the correlation between the age at surgery and number of pneumatized areas after surgery in the CC (**A**); *p* = 0.068, r = −0.344) and AC groups (**B**); *p* = 0.195, r = −0.228). Correlation between the age at surgery and difference between pre- and postoperative number of pneumatized areas in the CC (**C**); *p* < 0.001, r = −0.584) and AC groups (**D**); *p* = 0.148, r = −0.253). There was a negative correlation between the variables in the CC group (**A**,**C**).

Spearman’s rank-order correlation coefficient was employed to assess the correlation between the age at mastoidectomy and the number of air cell areas after surgery. A trend was observed in the CC group (Figure 3A, *p* = 0.068, r = −0.344), but not in the AC group (Figure 3B, *p* = 0.195, r = −0.228). Additionally, a correlation analysis was conducted for the changes (increments) in the number of air cell areas post-mastoidectomy (Figure 3C,D). A significant correlation was found between the postoperative increment of air cell area and the age at mastoidectomy in the CC group (Figure 3C, *p* < 0.001, r = −0.584), but not in the AC group (Figure 3D, *p* = 0.148, r = −0.253).

The correlation between the extent area number of cholesteatoma and postoperative number of pneumatized areas was analyzed using Spearman’s rank-order correlation coefficient. Despite the significant correlation between the extent area number of cholesteatoma and postoperative number of pneumatized areas in the CC group (r = −0.514, *p* = 0.004), no significant correlation was identified in the AC group (r = −0.116, *p* = 0.512).

Table 4 displays the pre- and postoperative pneumatization rates for each classified area. A Wilcoxon signed-rank test was conducted to compare the number of ears with air cells pre- and postoperatively in each classified area (Table 4). In the CC group, postoperative air cell development was observed in all areas except the anterolateral area of the peri-antrum. Conversely, in the AC group, air cell development was identified in the medial area of the peri-antrum and peritubal area, with no development in other areas. The rate of pneumatized area in the CC group was higher than that in the AC group, except for the posterolateral area of the peri-antrum preoperatively. Furthermore, when comparing classified areas, the pneumatized rate of the petrous apex area was the lowest in both CC and AC groups.

## 4. Discussion

Temporal bone pneumatization has been recognized as potentially inhibited by middle ear infections such as cholesteatoma, otitis media, and cholesterol granuloma [10,11,12]. Other influencing factors may include genetics, environment, nutrition, and various diseases [13,14,15]. Mastoidectomy could also impact temporal bone pneumatization. A previous study conducted experimental mastoidectomy in the tympanic bullae of piglets during pneumatization, revealing no air cell formation thereafter [16]. This led to speculation that mastoidectomy in childhood might halt subsequent pneumatization of the mastoid air cell system in humans [16]. Despite several CT-based observations assessing postoperative middle ear aeration, mostly in the short term (around one year) following CWU mastoidectomy [6,17,18,19], there has been a lack of studies utilizing human temporal bones to demonstrate postoperative pneumatization development after mastoidectomy. In this study, consecutive CT images from both short-term (one year) and long-term (over 5 years) studies were compared with preoperative images of patients successfully treated with CWU mastoidectomy for middle ear cholesteatoma. To the best of our knowledge, this is the first study identifying postoperative air cell development in children, although this study only included a group of patients with stable tympanic membrane after CWU surgery, thus, presumably having good tubal function.

The synthesis of air cells initiates with the formation of bone cavities, evolving from primordial bone marrow into mesenchymal tissue. Middle ear pneumatization is believed to result from mesenchymal thinning induced by a mesenchymal clearance factor of unknown origin and characteristics [20]. Essentially, pneumatization entails epithelium infiltrating developing bone and generating epithelium-lined air cell cavities. Pathological influences, including cholesteatoma, on middle ear mucosa during childhood may hinder pneumatization [3,21,22]. Therefore, preserving mastoid mucosa during mastoidectomy is presumed crucial to reactivating the subepithelial layer for air cell creation. Consistent with this concept, we have aimed to minimize mastoid opening just enough for cholesteatoma matrix removal transmastoidally.

Based on CT images with distinct preoperative benchmarks, postoperative air cell development occurred in ears with either CC or AC, five years or more following surgery. However, when comparing the number of pneumatized areas between the two groups, significantly better postoperative pneumatization was observed in the CC group than in the AC group (Table 3), despite the lack of significant differences in the extent of cholesteatoma between groups. Various etiological factors, including middle ear ventilatory conditions, may contribute to the difference in postoperative air cell development between the two groups. Dysfunction of middle ear ventilation at the levels of the eustachian tube and/or the tympanic isthmus is considered more attributable as a pathogenesis of AC [23,24] rather than that of CC, where the epithelial rest theory is widely accepted [25]. Pathological statuses of tympanic ventilation in children with AC might contribute to the suppression of middle ear pneumatization [3,26]. Another potential factor influencing the difference in postoperative air cell formation between the CC and AC groups is the age at mastoidectomy. Our study results indicated that the younger the age at first surgery, the greater the number of air cell areas found to develop postoperatively in the CC group (Figure 3A,C). This could partly be due to the younger average age at mastoidectomy in the CC group (6.2 ± 3.5 years) compared to the AC group (8.65 ± 3.62 years). Our findings also suggest that early surgical intervention for cholesteatoma removal helps minimize alterations to mastoid mucosa, positively influencing aeration and reactivation of the temporal bone pneumatization process when preserving mucosa during mastoidectomy or employing a “minimal” mastoidectomy approach. The mastoid cellular structure likely plays a critical role in stabilizing the aerated tympanic cavity while serving as an air reservoir [27,28] and facilitating transmucosal gas exchange function [29,30]. Although the impact of postoperative aeration on reducing the risk of retraction pocket formation and recurrence after CWU tympanomastoidectomy remains unclear, our results may justify the importance of maximal preservation of the mastoid mucosa when using CWU for pediatric cholesteatoma, at least from the perspective of postoperative temporal bone pneumatization.

This series identified some degree of air cell formation in each area by the time of the last postoperative CT examination: the periantral and peritubal areas in 80% to 90% for the CC group and 60% to 70% for the AC group; and the petrous apex area about 50% for the CC group and about 10% for the AC group. Postoperative air cell formation was not improved over the 5-year follow-up in the posterolateral area of the peri-antral cells because they were already formed at the time of the first surgery (Table 3). Air cell formations in these areas seem to follow the natural course of temporal bone pneumatization, developing over time until around puberty [31].

In contrast, the petrous apex cells, which had not yet formed at the time of surgery, showed a dramatic change in some cases postoperatively. Pneumatization of the petrous apex is known to be the final process of temporal bone air cell development [3,32]. In this study, the incidence of pneumatization of the petrous apex area in the CC group was 51.7%, consistent with normative data from maxillofacial CT, where 54% of patients showed pneumatization of the petrous apex to some degree [33]. Conversely, the low incidence of only 8.8% in the AC group is presumed to be related to the morphological characteristics of the protympanum in ears with AC [9]. Lee et al. (2015) reported that a well-pneumatized petrous apex was found less frequently in temporal bones with the protympanic structures typical for cholesteatoma cases [9,34].

Our study has several limitations. First, control cases without cholesteatoma and middle ear surgery were not included in this study because of the small number of cases. Second, it was limited to the observation of consecutive CT images, and no other clinical information beyond otoscopic findings at the time of CT examination was available. Third, the study focused on subjects with stable postoperative conditions, excluding patients with recurrent cholesteatoma. Fourth, we did not consider the possible effects of second-look procedures performed in some patients on further pneumatization processes. Fifth, due to the narrow space in temporal bone CT scans, a thorough assessment of the middle ear space was not feasible, potentially resulting in overlooked air cell structures. Sixth, since there are no reliable tests to evaluate eustachian tube function in children, the subjects of this study included only patients with a stable tympanic membrane postoperatively. To prove the impact of long-term postoperative development of pneumatization in children, further studies incorporating various factors such as eustachian tube function and the potential effect of tympanostomy tube insertion should be conducted.

## 5. Conclusions

We identified newly developed air cells in temporal bones after CWU mastoidectomy for pediatric cholesteatoma. A larger number of air cell areas were recognized in the CC group compared to the AC group. The younger the age at the first surgery, the greater the number of air cell areas found to develop postoperatively. Our results may justify the importance of preservation of the mastoid mucosa during mastoidectomy when the CWU technique is applied for CWU tympanomastoidectomy, at least for children of the younger age group and with CC, who may be likely to have a potential for subsequent development of air cell systems after surgery. However, further studies with larger sample sizes and assessments including cholesteatoma prognosis and functional outcomes are needed to corroborate these findings.

## Figures and Tables

**Table 1:** Inclusion and exclusion criteria.

Inclusion criteria	
(1)	Patients with middle ear cholesteatoma aged ≤15 years at the time of CWU tympanomastoidectomy
(2)	Patients with a successful otoscopic finding >5 years after surgery, i.e., stable tympanic membrane without perforation, middle ear effusion or retraction at the time of final CT examination
(3)	Patients with consecutive CT images scanned before and >5 years after surgery
Exclusion criteria	
(1)	Patients with any of the above CT images inappropriate for the evaluation of temporal bone air cell structures due to motion artifact or insufficient field of CT scanning

**Table 2:** Demographic data of the patients.

		CC Group	AC Group	*p*-Value
Number of patients		29	34	
Sex				
	Male	18 (62%)	23 (68%)	0.643
	Female	11 (38%)	11 (32%)	
Age at surgery		5 (2–15)	8 (2–15)	0.006
Duration of follow-up (month)		87 (47–173)	88 (48–346)	0.715
Stage of cholesteatoma (JOS criteria [8])				
	I	7 (24.1%)	6 (17.6%)	1.000
	II	21 (72.4%)	23 (67.6%)	0.786
	III	1 (3.4%)	5 (14.7%)	0.205
Cholesteatoma involvement in each site				
	P: Protympanum	13 (44.8%)	16 (47.1%)	1.000
	T: Tympanic cavity	29 (100%)	30 (88.2%)	0.118
	A: Attic	22 (75.9%)	29 (85.3%)	0.521
	M: Mastoid	16 (55.2%)	26 (76.5%)	0.108
Extent of cholesteatoma: total number of sites involved		2.76 ± 1.24	2.97 ± 1.09	0.617

CC: Congenital cholesteatoma, AC: Acquired cholesteatoma, JOS: Japan Otological Society.

**Table 3:** Comparison of the pneumatized area number between pre- and post-operation.

	Preop	Postop	*p*-Value
Total (n = 63)	2 (0–5)	4 (0–5)	<0.001
Congenital cholesteatoma (n = 29)	3 (0–5) *	4 (2–5) **	<0.001
Acquired cholesteatoma (n = 34)	2 (0–4) *	3 (1–5) **	0.003

* *p* < 0.05, ** *p* < 0.01.

**Table 4:** Comparison of the number of ears with air cells between pre- and post-operation in each area.

		Preop		Postop		*p*-Value *
Congenital cholesteatoma (n = 29)						
①	Anterolateral area of peri-antrum	26/29	(89.7%)	26/29	(89.7%)	1.000
②	Posterolateral area of peri-antrum	18/29	(62.1%)	26/29	(89.7%)	0.014
③	Medial area of peri-antrum	17/29	(58.6%)	28/29	(96.6%)	<0.001
④	Peritubal area	16/29	(55.2%)	25/29	(86.2%)	0.009
⑤	Petrous apex area	7/29	(24.1%)	15/29	(51.7%)	0.030
Acquired cholesteatoma (n = 34)						
①	Anterolateral area of peri-antrum	21/34	(61.8%)	21/34	(61.8%)	1.000
②	Posterolateral area of peri-antrum	24/34	(70.6%)	23/34	(67.6%)	1.000
③	Medial area of peri-antrum	11/34	(32.4%)	23/34	(67.6%)	0.007
④	Peritubal area	13/34	(38.2%)	24/34	(70.6%)	0.014
⑤	Petrous apex area	0/34	(0%)	3/34	(8.8%)	0.239

* Wilcoxon signed-rank test.

## Data Availability

All collected and analyzed data in this study are presented in this published article.
